# CBX7 is Dualistic in Cancer Progression Based on its Function and Molecular Interactions

**DOI:** 10.3389/fgene.2021.740794

**Published:** 2021-10-01

**Authors:** Jun Li, Taohui Ouyang, Meihua Li, Tao Hong, MHS Alriashy, Wei Meng, Na Zhang

**Affiliations:** ^1^ Department of the Second Clinical Medical College of Nanchang University, Jiangxi Province, China; ^2^ Department of Neurosurgery, the First Affiliated Hospital of Nanchang University, Jiangxi Province, China; ^3^ Department of Neurosurgery, Huashan Hospital of Fudan University, Shanghai, China; ^4^ Department of Neurology, the First Affiliated Hospital of Nanchang University, Jiangxi Province, China

**Keywords:** CBX7, cancer, molecular interaction, PRC1, RNAs

## Abstract

Chromobox protein homolog 7 (CBX7) is a member of the Chromobox protein family and participates in the formation of the polycomb repressive complex 1(PRC1). In cells, CBX7 often acts as an epigenetic regulator to regulate gene expression. However, pathologically, abnormal expression of CBX7 can lead to an imbalance of gene expression, which is closely related to the occurrence and progression of cancers. In cancers, CBX7 plays a dual role; On the one hand, it contributes to cancer progression in some cancers by inhibiting oncosuppressor genes. On the other hand, it suppresses cancer progression by interacting with different molecules to regulate the synthesis of cell cycle-related proteins. In addition, CBX7 protein may interact with different RNAs (microRNAs, long noncoding RNAs, circular RNAs) in different cancer environments to participate in a variety of pathways, affecting the development of cancers. Furthermore, CBX7 is involved in cancer-related immune response and DNA repair. In conclusion, CBX7 expression is a key factor in the occurrence and progression of cancers.

## Introduction

CBX7 belongs to the Chromobox proteins family and participates in the formation of the polycomb repressive complex 1 (PRC1). The polycomb group (PcG) proteins are transcriptional inhibitors that regulate several important developmental and physiological processes in cells (Levine et al., 2002). PcG proteins are originally identified in *Drosophila* as epigenetic transcriptional repressors (Histones are modified to cause transcription inactivation) expressed by homologous genes (Hox). Nowadays, they have been found in various metazoans and are highly conserved in evolution (Wang et al., 2015). Two subunit complexes in PcG proteins play an important role in epigenetic control: one is the initial inhibitory complex (PRC2), with enhancer of zeste homolog 2 (EZH2), EED, and SUZ12 as the main components and the other is the maintenance inhibitory complex (PRC1), with B-lymphoma Mo-MLV insertion region 1 (BMI-1), Chromobox 7 (CBX7) and E3 ubiquitin ligase RING1A/B as the main components (Schuettengruber et al., 2007; Scott et al., 2007; Wu et al., 2009). Functionally, in the PRC2 complex, EZH2 performs methyltransferase activity on lysine 27 on histone H3(H3K27) and is involved in the mono-, di-, and tri-methylation of lysine 27 on histone H3 (H3K27me1/2/3), which is essential for inducing transcriptional inhibition and stable gene silencing (Satijn et al., 2001; Geng and Gao, 2020). Conversely, in the PRC1 complex, CBX7 can bind to the H3K27me3 with its special domain (a highly conserved N-terminal chromodomain), thereby controlling the expression of multiple genes. In addition, the E3 ubiquitin ligase RING1A/B, a member of the PRC1 complex, is also recruited unto genes to promote histone H2A lysine 119 monoubiquitination (H2AK119ub1). These histone modifications induce chromatin compaction and aggregation, thereby inhibiting the transcriptional activity of target genes (Satijn et al., 2001; Ogawa et al., 2002; Gil et al., 2005; Wu et al., 2009; Kaito and Iwama, 2020). CBX7 inhibits genes expression at the transcriptional level in the nucleus.

Recent studies have shown that CBX7 not only regulates gene expression in the nucleus but also interacts with proteins involved in cell cycle regulation in the cytoplasm. The CBX7 protein can be divided into several subtypes, the classic ones being 36 KD protein CBX7 in the nucleus and 22KD protein CBX7 in the cytoplasm. Emerging evidence has shown that p22CBX7 rather than p36CBX7, inhibits cell proliferation, further demonstrating the complex mechanism of CBX7 regulating cell development (Cho et al., 2020). P22CBX7 in the cytoplasm may interact with some other proteins in the cytoplasm that play an important role in cell cycle progression to regulate cell proliferation, or that there is a reciprocal conversion that regulates cellular homeostasis in a complementary manner between CBX7 subtypes, thus forming negative feedback in CBX7 (Cho et al., 2020).

## Abnormal Expression of CBX7 in Cancer Progression

### Low Expression of CBX7 in Cancer Progression

#### Breast Cancer

Bioinformatic analysis of Chromobox proteins family has shown that the most significant difference between breast cancer and normal mammary tissue is the low mRNA expression of CBX7 (Li X. et al., 2020). One study of breast cancer implied that patients with low CBX7 had lower survival and were more likely to develop lymph node metastasis, P53 mutations, and the cancer further deteriorated, metastasized over time (Li X. et al., 2020). The mechanism for its carcinogenicity may be that CBX7 acts as a novel epigenetic regulator of the *Wnt*/β-catenin pathway in breast cancer to determine cancer progression (Li X. et al., 2020). In breast cancer, CBX7 increases Dickkopf-1 (DKK-1, a *Wnt* antagonist) gene transcription, thus indirectly affecting the *Wnt* signaling pathway (Bafico et al., 2001; Kim et al., 2015). For transcriptional activation of the DKK-1 gene, CBX7 can directly interact with p300 acetyltransferase and recruit p300/CREB binding protein (CBP) to the DKK-1 promoter, thereby inhibiting histone deacetylases (HDAC)-mediated histone deacetylation (Kim et al., 2015). In breast cancer, due to the lack of CBX7, the transcriptional activation complex p300/CBP dissociates from the promoter region of DKK-1 and restarts HDAC-mediated DKK-1 gene silencing. The decreased expression of *Wnt* antagonist DKK-1 can reactivate the *Wnt*/β-catenin/T-cell factor (TCF) pathway, leading to nuclear translocation of β-catenin and up-regulation of the expression of TCF target genes including *C-MYC* (Wang et al., 2008; Kim et al., 2015). Therefore, CBX7 plays a cancer-inhibiting role by guiding the synthesis of DKK-1 to attenuate the *Wnt* pathway in breast cancer cells. In addition, there is evidence that CBX7 also plays an important role in controlling glucose metabolism in breast cancer cells (Iqbal et al., 2021). mTORC1 signaling is a known determinant of cancer metabolism and frequently deregulated in breast cancer (Creighton, 2007; Mossmann et al., 2018). The increase of glycolysis induced by silencing CBX7 may imply that CBX7 regulates the mTORC1 pathway to control aerobic glycolysis in breast cancer (Iqbal et al., 2021). Both Chromobox 2 (CBX2) and CBX7 can be used as subunits of PRC1 to act as a reader to recognize H3K27m3, thus hindering the transcription of INK4a/ARF tumor suppressor genes (Jangal et al., 2019). Interestingly, CBX2, which is homologous to CBX7, has a high content in breast cancer (Liang et al., 2017). However, CBX7 content in breast cancer is often very low, indicating that CBX7 plays its other tumor suppressor role independently of PRC1. The reason why CBX7 is lost in breast cancer cells may be ascribed to lncRNA NEAT1 targeting CBX7 (Yan et al., 2020). It has been determined that the protein level of CBX7 is positively correlated with NEAT1 in breast cancer cells, proving that CBX7 is indeed a target gene regulated by NEAT1 (Yan et al., 2020). Moreover, miR-181b induced by high mobility group AT-hook 1 (HMGA1) interferes with CBX7 mRNA at the translation level to inhibit its expression, resulting in a lack of CBX7 in breast cancer (Mansueto et al., 2010). In these ways, CBX7 acts as an intermediate in breast cancer, creating a series of chain reactions.

#### Liver Cancer

The mRNA expression of CBX7 increases with liver development after birth and is maintained at a normal level to regulate the epigenome, transcriptome, and liver function (Lu et al., 2012). Accumulative evidence has shown that CBX7 content is significantly reduced in liver cancer tissues (Lu et al., 2012). In liver cancer, the expression of CBX7 mRNA is the highest in grade Ⅰ tumors among grade classification of tumor. With the increase of tumor grade, the expression of CBX7 mRNA shows a downward trend (Ning et al., 2018). In addition, the decreased expression of CBX7 is significantly correlated with liver cirrhosis by the chi-square test (Zhu et al., 2019). In patients with hepatocellular carcinoma, the high expression of CBX7 is related to a better survival rate of patients (Ning et al., 2018). One of the proposed mechanisms for cancer suppression is that CBX7 exerts a cancer suppressor effect by inhibiting the expression of cyclin E (Forzati et al., 2012). CBX7 and HDAC2 combine to form a complex and are fixed on the promoter of CCNE1, thereby repressing its transcriptional activity. The study suggested that overexpression of CBX7 would interfere with the composition of PRC1. In addition, it was proven that CBX7 dose-dependently reduced the transcriptional activity of the CCNE1 promoter (Forzati et al., 2012). We postulate that CBX7 exerts special effects independently of PRC1. On the contrary, the HMGA1b protein is a competitor of this action. The antagonistic effect of CBX7 versus HMGA1b protein on the CCNE1 promoter could inhibit cell proliferation and migration (Fusco and Fedele, 2007; Pallante et al., 2010; Forzati et al., 2012). For CBX7 itself, its expression may be regulated by non-coding RNA, such as miR-181 positively regulated by HMGA1. it may combine with the 3′-UTR of CBX7 mRNA to limit its translation (Forzati et al., 2014). Similarly, the mode of action of CBX7 in liver cancer is a continuous one-way pathway. CBX7 not only controls the synthesis of related proteins but also is restricted by other molecules.

#### Colonic Cancer

CBX7 is associated with multiple clinicopathologic parameters in colon cancer. Compared with normal colonic mucosa, CBX7 expression is reduced or absent in a large number of colon cancer specimens, and the absence of CBX7 expression is remarkably correlated with the poor prognosis of colon cancer patients (Pallante et al., 2010). The mRNA level of CBX7 in colon cancer samples is lower than that in normal colonic tissues, suggesting that the CBX7 expression is inhibited at the transcriptional level (Pallante et al., 2010). However, the mechanism by which it is suppressed at the transcriptional level remains unclear. Furthermore, CBX7 negatively regulates cyclin E which is involved in a G1-S phase transition. CBX7 is positively correlated with E-cadherin expression (Federico et al., 2009; Pallante et al., 2010). However, two components of the PRC1 which includes CBX7 and BMI-1 regulate the expression of E-cadherin differently. Studies had shown that BMI-1 significantly down-regulates the content of E-cadherin in colon cancer and promotes the epithelial-mesenchymal transition (EMT) process (Zhang et al., 2016). The different regulatory effects on the same protein between the two reveal that CBX7 may be independent of PRC1 composed of BMI-1 to achieve EMT inhibition. Not surprisingly, like CBX7 in breast cancer, abnormal activation of the *Wnt* signaling pathway is found in 90% of colon cancer, and one of the reasons may be the absence of secreted frizzled-related protein 4(sFRP4) (Liu et al., 2020). The sFRP4 could compete with *Wnt* proteins *via* binding their receptor (Frizzled) to act as an antagonist of the *Wnt* signaling pathway in colonic cancer (Liu et al., 2006; Liu et al., 2020). Interestingly, both DKK-1 and sFRP4 are antagonists of the *Wnt* signaling pathway, but the effect of CBX7 in regulating sFRP4 in colon cancer is quite different from the effect of regulating DKK-1 in breast cancer. CBX7 and other PcG proteins, such as EZH2 and Jumonji, AT rich interactive domain 2 (JARID2) are found to be enriched in the SFRP4 gene promoter region to regulate gene expression without DNA methylation (Liu et al., 2020). Perhaps it is the synergistic effect of CBX7, EZH2, and JARID2 on histone methylation modification that leads to epigenetic gene silencing (Walters et al., 2014; Pallante et al., 2015). Therefore, silencing the sFRP4 gene indirectly enhances the *Wnt* pathway, thereby promoting the development of cancer. The contradiction between the two roles of CBX7 in colon cancer further suggests that multiple CBX7 regulatory pathways may exist. In cancer progression, different cancer-promoting or anti-cancer pathways have gained corresponding advantages due to their expression intensity. It would be beneficial to further actively explore the pathways of CBX7 involvement.

#### Thyroid Cancer

It has been reported that the loss of CBX7 is associated with a highly malignant phenotype in thyroid cancer (Pallante et al., 2008). CBX7mRNA is high in most of follicular thyroid adenomas and rarely develops into cancer (Pallante et al., 2008; Monaco et al., 2014). However, a low level of CBX7 is shown in thyroid cancer. For example, Hurtle adenoma is a rare differentiated thyroid tumor with the highest incidence of metastasis, and as the tumor worsens, CBX7 gradually decreases at the transcriptional level and the tumor develops into Hurtle carcinoma (Cheung et al., 2000; Monaco et al., 2014). The decrease of CBX7 in thyroid cancer may be due to the negative regulation of tumor protein HMGA1 (Chiappetta et al., 1995). A loss of epithelial features and an acquisition of mesenchymal phenotypes are signs of tumor aggressiveness, which are often related to a lack of E-cadherin (Mansueto et al., 2010). It has been confirmed that CBX7 can actively regulate the expression of E-cadherin by interacting with HDAC2 and inhibiting its effect on the E-cadherin promoter (Federico et al., 2009). In addition, HDAC2 catalyzes the acetyl transfer of core histones and is generally considered to be a transcriptional repressor due to its ability to induce gene silencing (Federico et al., 2009; Mansueto et al., 2010). In thyroid cancer, H3k27me3 catalyzed by EZH2 is overexpressed (Tsai et al., 2019). However, CBX7, which has the recognition function of H3k27me3, did not follow its increasing trend to show high expression. This indicated that CBX7 did not act as a member of PRC1 to link up EZH2-mediated H3k27me3 to assist gene silencing. Instead, CBX7 can up-regulate the expression of FOS, FOSB, and EGR1, which are mostly involved in the physiological process of inhibiting cancer (Pallante et al., 2014). The exact molecular mechanism of promoting the expression of these genes is unclear. There is clear evidence that CBX7 is associated with osteopontin protein, which is encoded by the SPP1 gene (Sepe et al., 2015). Osteopontin is a well-known protein involved in cancer progression by promoting cell invasion and migration, leading to tumor metastasis (Rohde et al., 2007). On the one hand, CBX7 regulates cell migration by blocking HMGA1b and inhibiting SPP1 gene expression; on the other hand, CBX7 and HMGA1b limit SPP1 gene expression by regulating the activity of nuclear factor kB (NF-κB) (Sepe et al., 2015).

#### Glioma

In gliomas, unsurprisingly, CBX7 and HDAC2 jointly address the CCNE1 gene promoter induced G1/S phase arrest, which is similar to the effect of CBX7 in liver cancer, and CBX7 also enhances the expression of DKK-1 by binding to the DKK-1 promoter, which is similar to the effect of CBX7 in breast cancer (Mansueto et al., 2010; Kim et al., 2015; Bao et al., 2017; Yu et al., 2017). Not only that, there is a strong connection between CBX7 and the Hippo signaling pathway in gliomas (Nawaz et al., 2016). CBX7 inhibits YAP/TAZ, down-regulates CTGF (adverse prognostic product of tumor) with PRC2 as a member of PRC1, and reduces the phosphorylation level of c-Jun NH2-terminal kinase (JNK) (Nawaz et al., 2016). Among these, connective tissue growth factor (CTGF) plays an auxiliary role in gliomas by activating the ITGB1-TrkA-NF-kB pathway, which can inhibit E-cadherin at the transcription level and enhance the invasion and migration of glioma cells (Edwards et al., 2011). Thus, CBX7 indirectly inhibits the decrease of E-cadherin, plays an important role in its quantity homeostasis. For CBX7 expression, the orphan nuclear receptor TLX (NR2E1, a transcription repressor) is related to the regulation of CBX7 expression (OʼLoghlen et al., 2015b). The ectopic expression of NR2E1 binds to the CBX7 locus, activates CBX7 expression. In contrast, CBX7 binds to the NR2E1 locus as a member of the PRC1 and inhibits it as part of a regulatory feedback loop (OʼLoghlen et al., 2015b). These also imply that different pathways can also influence each other to regulate cancer progression.

#### Pancreatic Cancer

In pancreatic cancer, the expression of CBX7 is lower than that of normal pancreatic tissues (Ni et al., 2017). Microarray and GO-pathway analysis predict that CBX7 promotes the activation of Phosphatase and tensin homolog (PTEN) and then inhibits the downstream phosphatidylinositol 3-phosphate kinase (PI3K)/AKT pathway (Ni et al., 2017). Interestingly, the mechanism of increasing PTEN transcription may be the same as the enhanced DKK-1 expression in breast cancer, where CBX7 recruits p300 independently of PRC1 to the promoter region to participate in epigenetic changes (Kim et al., 2015; Ni et al., 2017). It is well known that PTEN is a classic tumor suppressor (Ying et al., 2011). Once PTEN is abnormally expressed, it will lead to the activation of the PI3K/AKT signaling pathway, then target NF-kB and C-MYC transcription factors, which are conducive to the survival and development of cancers (Asano et al., 2004; Ying et al., 2011).

### High Expression of CBX7 in Cancer Progression

#### Gastric Cancer

In gastric cancer, CBX7 is highly expressed, contrary to its low expression in all of the above-mentioned cancers. It has been proven that CBX7 acts as a carcinogen mainly by inducing the down-regulation of p16^INK4a^/ARF, which is blocked by the BMI-1 protein (Guo et al., 2007; Zhang et al., 2010). Besides, accumulated evidence shows that CBX7 has a similar function to BMI-1 in gastric cancer, suggesting that CBX7 may participate and function in a way related to BMI-1 regulation (Guo et al., 2007; Ni et al., 2018). Although multivariate Cox proportional risk model analysis shows that CBX7 is not an independent prognostic factor for gastric cancer deterioration (Zhang et al., 2010), the carcinogenesis role of CBX7 should not be underestimated. In gastric cancer, CBX7 actively regulates the stem cell characteristics of gastric cancer cells through the AKT-NF-kB-miR-21 pathway (Ni et al., 2018). Therefore, CBX7 utilizes at least the two pathways mentioned above to play a cancer-promoting role in gastric cancer (Zhang et al., 2010; Ni et al., 2018). It is worth considering that miR-21 in the microRNA family is a downstream target of CBX7. However, in the presence of miR-421 inhibitors, the growth of gastric cancer cells is inhibited. Therefore, miR-421 may act as an upstream molecule to regulate the target CBX7, suggesting a deeper link between CBX7 and the microRNA family (Jiang et al., 2010; Ni et al., 2018).

#### Prostatic Cancer

**FIGURE 1:**
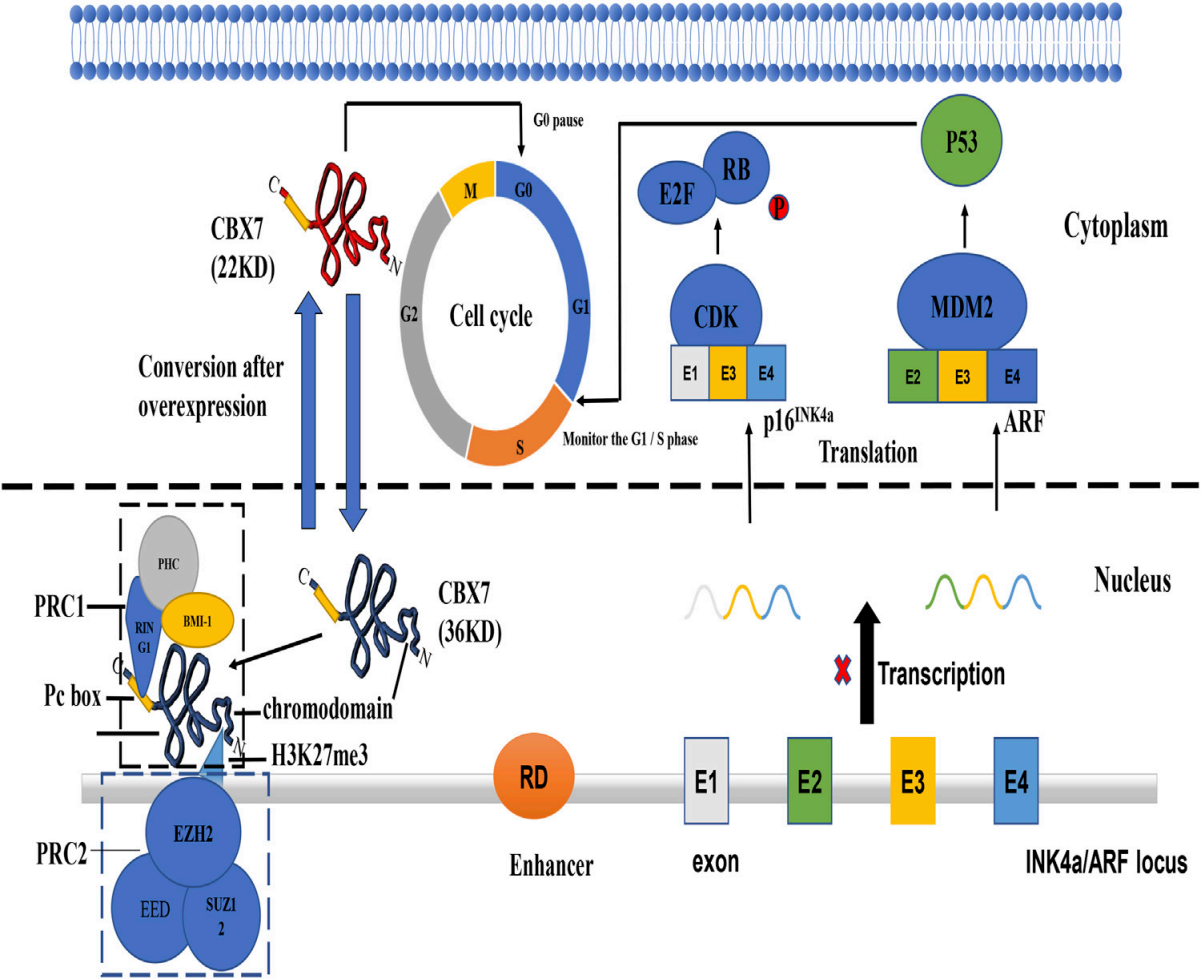
The mechanism of tumor suppressor gene *INK4a/ARF* and functions of different CBX7 isoforms. (Kim and Sharpless, 2006) INK4a/ARF can express two co-exon products p16 and ARF. P16 ^INK4a^ can inhibit the phosphate of RB protein by interacting with cycle-dependent kinase. Inactivation of CDK4/6 promotes Rb/E2F1 association triggering G1/S transition. ARF can bind to Mouse Double Minute 2 protein (MMD2) protein to inactivate it, thereby releasing its inhibitory effect on p53 protein. P36CBX7 inhibits the expression of ARF in the nucleus to cause p53 protein abnormalities and make the cell cycle out of control at the G1/S regulatory point. P22CBX7 interacts with related proteins in the cytoplasm to keep the cell cycle in the G0 phase. Both have opposite effects on cell proliferation, showing the duality of CBX7.

In prostate cancer, CBX7 is also positively correlated to cancer progression (Bernard et al., 2005). Overexpression of CBX7 contributes to the down-regulation of the expression of the tumor suppressor gene *INK4a/ARF* (Figure 1) (Bernard et al., 2005). Generally, androgens are favorable factors for prostatic cancer development, but CBX7 plays an important role in maintaining prostate cancer cells growth by cooperating with C-MYC with androgen-independent transformation (Bernard et al., 2005). Recently, it has been reported that GOLPH3 can bind to the CBX7 protein in prostatic cancer to promote cell proliferation and inhibit cell apoptosis (Gong et al., 2020). More importantly, circGOLPH3, like most of circRNAs, has abundant microRNA binding sites (Gong et al., 2020). CircGOLPH3 may have a “sponge effect”, absorbing microRNAs and reducing the number of microRNA bound to target mRNA, thus increasing the expression level of target protein such as CBX7 (Gong et al., 2020). While this clearly shows the interaction in RNAs to regulate CBX7, for the binding between GOLPH3 and CBX7, it is questionable whether circGOLPH3 can also directly regulate the activity of CBX7 protein in post-translational modification.

#### Lymphoma

Finally, CBX7 is also highly expressed in lymphomas. CBX7 promotes T-cell lymphoma, and when sensitized oncogenes are directed to B-cell loci, CBX7 also promotes aggressive B-cell lymphoma, with a presence and latency similar to that of BMI-1 (Scott et al., 2007). CBX7 may cause the occurrence of lymphoma by enhancing stem cell self-renewal or increasing the replication potential of cancer stem cells (Scott et al., 2007). Although CBX7 may affect the transcription of multiple genes, inhibiting *INK4a/ARF* seems to be more important for its carcinogenic potential (Scott et al., 2007). However, CBX7 does not regulate cancer progression alone, the long latency and incomplete penetrance observed after overexpression of CBX7 suggest that aberrant CBX7 expression is not sufficient for causing lymphoma. CBX7 usually cooperates with C-MYC in the formation of aggressive lymphomas during lymphocyte genesis (Scott et al., 2007). More commonly, CBX7 is co-expressed with ZBTB7 in human follicular lymphoma, increasing the possibility that these proteins collectively control *INK4a/ARF* expression (Maeda et al., 2005). It also should be noted that CBX7 often works with other proteins, such as B-cell lymphoma 2(BCL-2), to accelerate the progress of lymphoma (Ramaswamy et al., 2001; Scott et al., 2007). Therefore, CBX7 could work with different partner molecules to produce different effects (pro-cancer or anti-cancer). Determining the specific molecular mechanism of CBX7 is very interesting and deserves further study.

## CBX7 Interacts With Different RNAs in Different Cancer Environments

### Interaction of CBX7 With microRNAs

MicroRNAs have obvious tissue specificity and can interact with CBX7 to affect cancer progression (Correia de Sousa et al., 2019). Many members of the microRNA family are involved in interference with CBX7 expression. For example, luciferase reporter assay has shown that miR-19 promotes the proliferation, migration of lung cancer cells by binding to the 3′-UTR of CBX7 mRNA and inhibits CBX7 expression at the translation level (Peng et al., 2018). Similarly, in lung adenocarcinomas, miR-181 overexpression promotes epithelial-mesenchymal transition (EMT) by directly targeting CBX7 (Pei et al., 2020). In addition, miR-375 plays a cancer-promoting role in prostate cancer by influencing the epigenetic regulation of transcriptional programs through its ability to directly target the polycomb complex member CBX7 (Pickl et al., 2016). Moreover, compared with CBX7+/+MEFs(mouse embryonic fibroblasts), CBX7−/−MEFs show a higher level of miR-181 in breast cancer while CBX7 ± MEFs express a moderate level, suggesting that CBX7 negatively regulates the expression of miR-181 (Pallante et al., 2015). Therefore, on the one hand, CBX7 can negatively regulate the expression of miR-181. On the other hand, since miR-181 binds to the mRNA 3′-UTR of CBX7, the expression of CBX7 is restricted, thus forming a synergistic cycle that promotes cancers (Pallante et al., 2015; Peng et al., 2018; Yan et al., 2020). MiR-155 is positively regulated by CBX7 in MEFs and colon carcinomas. Moreover, it has been determined that K-ras is a target of miR-155, the overexpression of miR-155 causes a sharp drop in K-ras mRNA and protein levels (Forzati et al., 2017). In glioma, the *in vitro* and *in vivo* functional assays results indicate that overexpression of miR-18a induced by miR-18a mimics may promote cell proliferation and migration of liver cancer cells by acting on CBX7 (Wu et al., 2017). On the contrary, miR-18a shows a cancer suppressor effect in primary ovarian tumors, due to the dual role of CBX7 as a bridge molecule in different cancers progression (Zhao et al., 2020). In addition, it has been reported that miR-9 can induce cell senescence without affecting the expression of CBX7, but the expression of CBX7 weakens its ability to induce senescence, which may be related to the regulation of senescence involved in p16^INK4a^ (OʼLoghlen et al., 2015a).

### Interaction of CBX7 With Long Noncoding RNAs

There are few studies on the regulatory relationship between long noncoding RNA lncRNA and CBX7. Some lncRNAs can directly or indirectly influence the role of CBX7 in cancer progression. In breast cancer, lncRNA NEAT1 is highly expressed in breast cancer tissues and closely related to clinical stage, lymph node metastasis (Li et al., 2017; Yan et al., 2020). It has been reported that with the down-regulation of NEAT1, the expression of CBX7 protein in breast cancer cells is also significantly down-regulated (*p* < 0.01) (Yan et al., 2020). lncRNA NEAT1 is mainly located in the cytoplasm but also exists in the nucleus. It is speculated that lncRNA NEAT1 may play a role in regulating CBX7 expression in terms of chromatin status, DNA binding, or the fate of newly transcribed mRNA in the nucleus (Yan et al., 2020). lncRNA ANRIL can coordinate or reverse the “reader” function of CBX7 at the transcriptional level (Yap et al., 2010). The p16 gene and the gene transcribed into ANRIL are located on the same chromosome, and CBX7 chromodomain uses distinct regions and residues for binding H3K27me or lncRNA (Pasmant et al., 2007; Yap et al., 2010). Both PRC1 and PRC2 are retained at a repression site of a target gene through their association with the nascent ANRIL transcripts of Pol II, ANRIL/CBX7 binding could result in dissociation of PRC1 from H3K27me, leading to a reversal of transcriptional repression of the target gene (Yap et al., 2010). In addition, lncRNA SNHG7 can also indirectly influence the expression of CBX7 by regulating miR-181 in lung adenocarcinoma (Pei et al., 2020). It can be seen that the interaction between CBX7 and lncRNA family is not one-to-one, but a complex regulatory network between lncRNA and microRNA families.

### Interaction of CBX7 With Circular RNAs

CircRNA has been proved to be an important molecule that can be used as a therapeutic target for tumors. However, studies on the relationship between circRNA and CBX7 are few and currently limited to prostate cancer (Chen et al., 2019; Gong et al., 2020). In the study of prostate cancer, it is found that CBX7 can specifically bind to circGOLPH3 based on the results of mass spectrometry, but the detailed mechanism of circRNA-CBX7 binding remains unclear (Gong et al., 2020). The answer to the direct and specific binding of GOLPH3 to CBX7 may be that the two directly bind to prevent CBX7 degradation. Moreover, in prostate cancer, circCSNK1G3 promotes cell growth by interacting with miR-181 involved in the regulation of CBX7 (Chen et al., 2019; Pei et al., 2020). Of note, circRNAs can bind, store, classify and isolate proteins to specific subcellular locations (Huang et al., 2020). Given that the above discussion in various cancers, CBX7 mainly functions as an epigenetic regulator in the nucleus. Therefore, it is possible to speculate whether CBX7 can be dispersed into different subcellular locations under the action of circular RNAs to show a confusing result, which is mistaken for the duality of CBX7. For example, CBX7 is highly expressed in cells, however, the amount of CBX7 in the nucleus is small and its regulatory role is not significant. The relationship between circRNA family and CBX7 may further uncover the complex mechanism of CBX7.

## The Relationship Between the Physiological Function of CBX7 and Cancer

### CBX7 and the Immune Response

It has been reported that CBX7-deficient CD4+T cells express more FasL and display the FasL gene promoter demethylation after antigen-specific TCR activation (Li et al., 2014). It is suggested that CBX7 can inhibit the expression of FasL in CD4+ T cells, resulting in significantly reduced demethylation of the FasL gene promoter. It is well known that the Fas-FasL association is a typical pathway that induces T cell apoptosis (Fisher et al., 1995). Therefore, CBX7 can inhibit activation-induced T cell apoptosis, indicating that it plays a role in the adaptive immune response. Interestingly, a small proportion of people infected with HIV remain asymptomatic and maintain a high CD4 + T cell count after years of seroconversion (Imami et al., 2013). A meta-analysis showed that the CBX7 gene is highly correlated with viral control (Ding et al., 2019). In addition, changes in somatic cell copy numbers of the CBX family inhibit the infiltration of immune cells in melanoma (Li D. et al., 2020). The analysis of immune infiltration for the CBX family showed that the expression of CBX7 is related to the abundance of these six immune cells (B cells, CD8+ T cells, CD4+ T cells, macrophages, neutrophils, and dendritic cells) (Li D. et al., 2020). Moreover, in prostate cancer, C-C motif chemokine ligand 2 (CCL2) is a main target of PRC1, and the H3K27me3 inhibitory marker that is bound with CBX7 is relatively low in the promoter region of the CCL2 gene, which leads to massive production of CCL2 (Su et al., 2019). The cytokine CCL2 not only promotes self-renewal by binding to C-C motif chemokine receptor 4 (CCR4) on prostate cancer cells but also recruits tumor-associated macrophages and Tregs in cancer metastasis sites by paracrine, thus creating a profound immunosuppression and angiogenesis microenvironment, confirming once again the importance of CBX7 in tumor immunity (Josefowicz et al., 2012; Su et al., 2019).

Immune cells infiltrating in the tumor microenvironment can regulate cancer progression, which is the current trend of cancer therapy (Quail and Joyce, 2013). CBX7 can play its duality in different cancer tissues, but it could be beneficial to the immune system of the human body. It would be interesting to assess whether CBX7 can be used as an intermediate molecule between cancers and immunity.

### CBX7 and DNA Damage

It is well known that the source of cancers can be naturalized with proto-oncogene mutations and cancer suppressor gene deletions. The fundamental reason for cancer lies within the problems within DNA damage and repair (OʼConnor, 2015). Recently, the contact between CBX7 and DNA damage repair has emerged in cancers. In melanoma, the downstream kinase targets of CBX7 are Polo-like kinase 1(PLK1) and cyclin-dependent kinase 1(CDK1), which are related to genome stability. Therefore, CBX7 is indirectly involved in DNA damage repair (Li D. et al., 2020). Moreover, in melanoma studies, it has been shown that in the presence of CBX7 inhibitors, DNA damage repair is inhibited (Connelly et al., 2016). In addition, RING1, a member of the PRC1, has been shown to possess ubiquitin ligase activity toward histone H2A, suggesting that the recruitment of polycomb proteins may be responsible for the ubiquitination of histone H2A at DNA lesions. CBX7 is also a member of the PRC1. In other words, CBX7 may locate at DNA damage sites with the recruitment of PRC1 containing RING1 (Chou et al., 2010; Pei et al., 2020). All these suggest the importance of CBX7 in DNA damage repair.

## Discussion

Clinically, the expression of CBX7 in different cancers is inconsistent for cancer progression, showing double-sidedness. CBX7 is lowly expressed in cancers of the breast, pancreas, liver, thyroid, colon, and glioma, but highly expressed in cancers of the stomach, prostate, and lymph. We outline above that CBX7 may regulate cell proliferation, apoptosis, and metastasis through different targets in different tissues. Based on the circumstances regarding CBX7 in cancers, it may be bold to speculate that the double-sidedness of CBX7 in cancer progression is closely related to its regulatory pathways (Table 1). Limited by our understanding of various cancer pathways, we describe some of the regulatory pathways in which CBX7 participates in cancers.

**TABLE 1:** CBX7 participates in various pathways and plays different physiological effects in different cancers.

Cancer types	Main pathway of participation	Effect	Result	References
Breast	Wnt pathway	Inhibit DKK-1 genomic protein deacetylation	Inhibit the self-renewal ability of breast cancer cells	Kim et al. (2015)
Liver	—	Bind to the CCNE1 promoter with HDAC2	Inhibit cell proliferation and migration	Forzati et al. (2012)
Colon	Wnt pathway	Combine sFRP4 promoter to methylate to silence genes	Promote cancer cell proliferation	Liu et al. (2020)
Pancreas	PI3K/AKT	Recruit P300 to promote the activation of PTEN	Induce apoptosis	Ni et al. (2017)
Thyroid	—	Regulate the expression of E-cadherin and osteopontin	Maintain epithelial characteristics and regulate cell migration	Federico et al. (2009); Sepe et al. (2015)
Glioma	Hippo pathway	Down-regulate the decrease in E-cadherin caused by CTGF	Prevent the invasion and migration of glioma cells	Nawaz et al. (2016)
Stomach	AKT—NF-kB—miR-21	Promote the activation of AKT and ERK	Actively regulate the stem cell characteristics of gastric cancer cells	Ni et al. (2018)
Prostate	INK4a/ARF	Epigenetic suppression of tumor suppressor gene	Maintain cell proliferation and growth	Bernard et al. (2005)
Lymph	INK4a/ARF	Epigenetic suppression of tumor suppressor gene	Enhance the replication potential of cancer stem cells	Scott et al. (2007)

**FIGURE 2:**
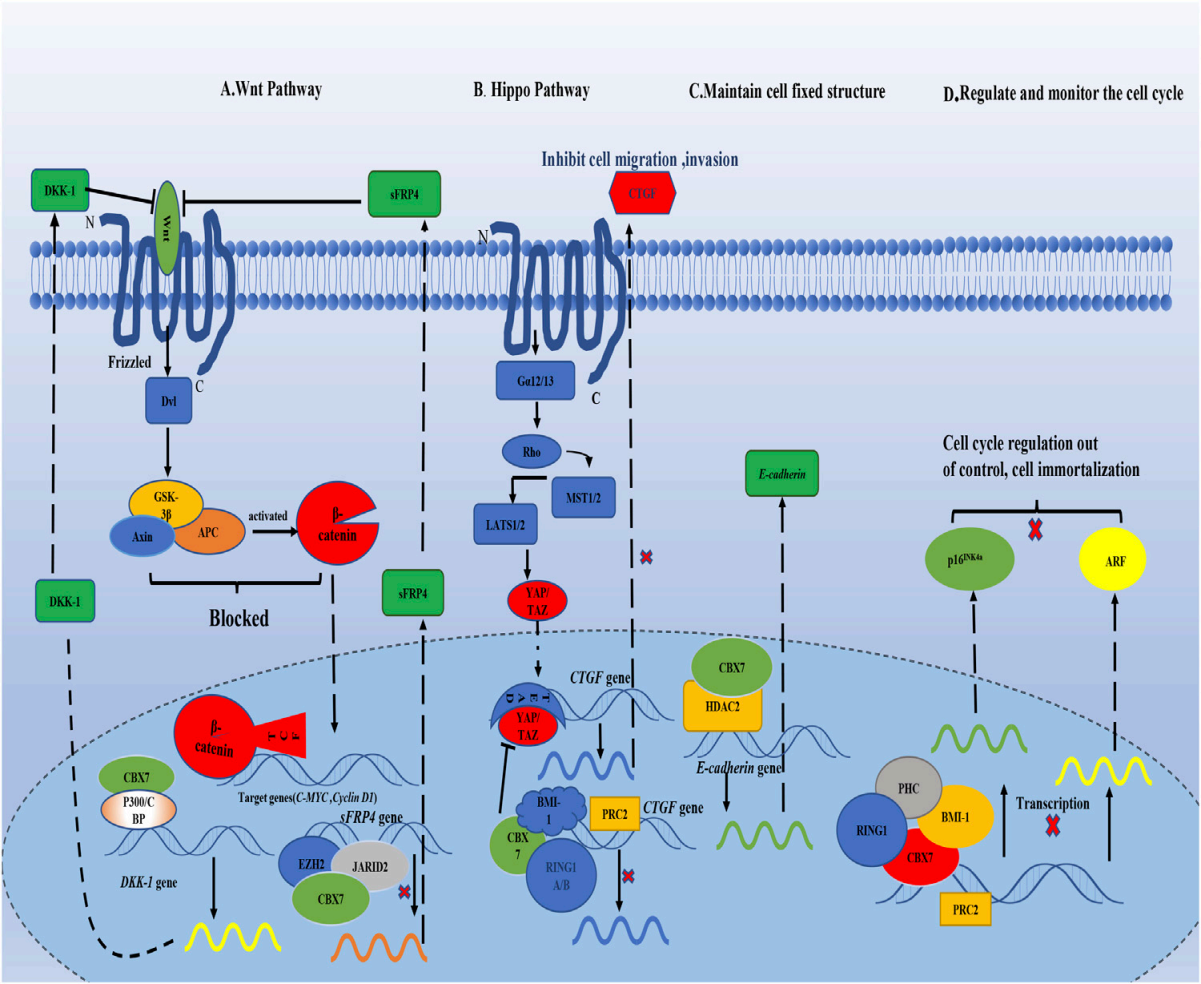
CBX7 interacts with proteins in the nucleus, transcriptionally controls the synthesis of related proteins, and regulates different signaling pathways. **(A)** CBX7 enhances the expression of Wnt antagonist DKK-1 by interacting with other molecules in epigenetics, leading to the blockage of downstream signal transduction of Wnt pathway, and then the expression of C-MYC and Cyclin D are restricted to maintain normal cell proliferation. But CBX7 inhibits the expression of sFRP4, which is also a Wnt antagonist.**(B)** On the one hand, CBX7 directly inhibits CTGF expression epigenetically; on the other hand, CBX7 antagonizes YAP/TAZ to indirectly restrict CTGF expression, which inhibits cell invasion and migration. **(C)** CBX7 cooperates with HDAC2 to promote the synthesis of E-cadherin in transcription to maintain cell fixed structure. **(D)** CBX7 relies on PRC1, and cooperates with PRC2 to epigenetically inhibit the expression of INK4a/ARF, making cell proliferation out of control.

**FIGURE 3:**
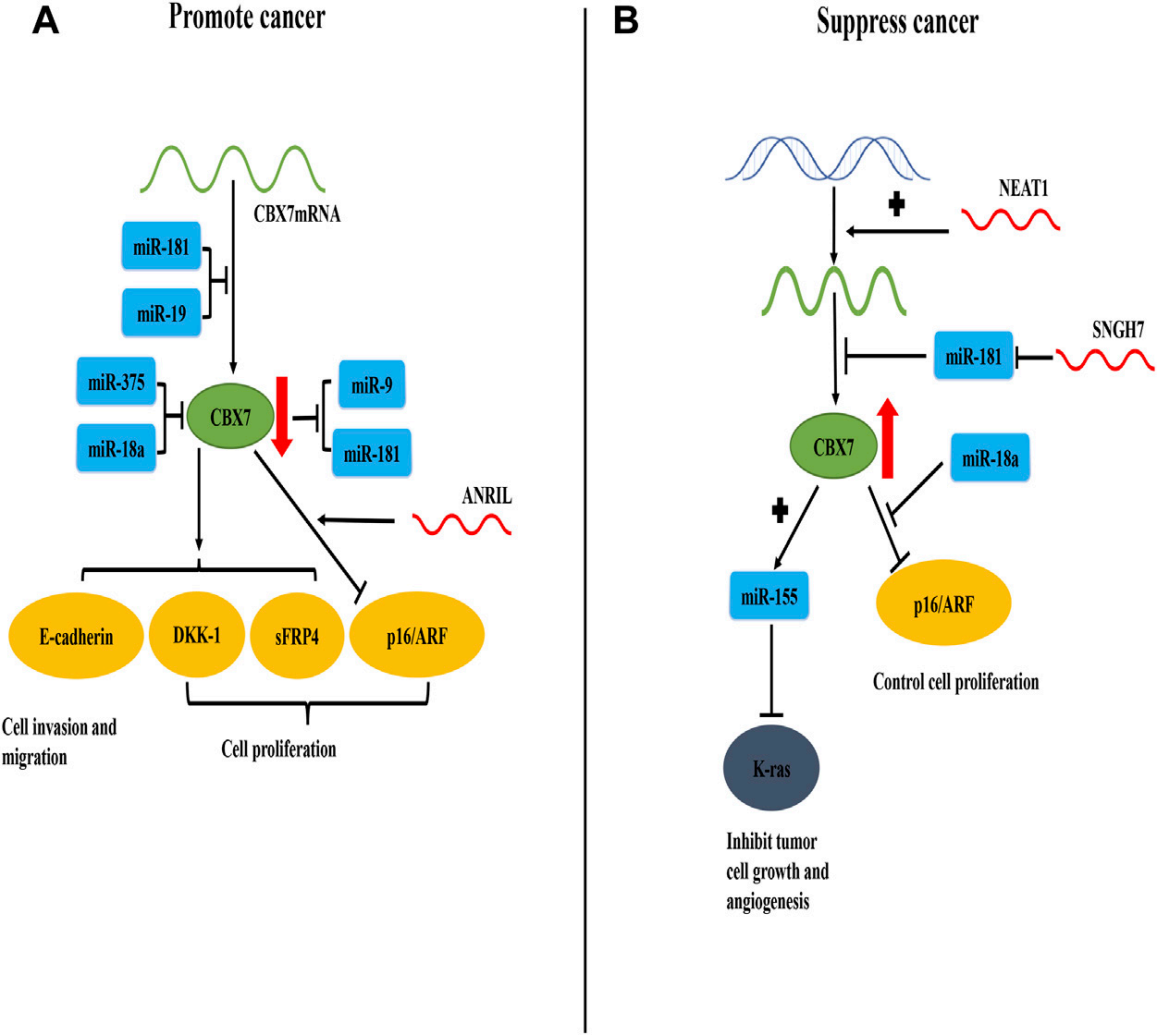
CBX7 expression is regulated by RNA molecules and the interaction between CBX7 and RNA molecules promotes or inhibits cancer progression. **(A)** In terms of quantity, miR-181 and miR-19 combine with the 3′-UTR of CBX7 mRNA to inhibit its translation and reduce its content. In terms of function, miR-375 and miR-18a act on CBX7 protein to decrease its activity. Therefore, the expression of DKK-1, sFRP4 and E-cadherin mediated by CBX7 decreases, resulting in uncontrolled cell proliferation, cell migration and invasion. CBX7 can also strengthen the inhibition of INK4a/ARF expression under the action of ANRIL, leading to uncontrolled cell cycle regulation and cell proliferation. Meanwhile, CBX7 can also inhibit miR-9 and miR-181. **(B)** In terms of quantity, NEAT1 promotes the synthesis of CBX7 at the transcription level, and SNGH7 indirectly facilitates the synthesis of CBX7 protein by blocking miR-181 at the translation level. Therefore, high content of CBX7 protein can inhibit abnormal K-ras protein *via* miR-155, thereby controlling tumor cell growth and angiogenesis. Meanwhile, miR-18a can relieve the transcriptional inhibition of INK4a/ARF by CBX7, thereby restoring normal cell proliferation.

In general, the current consensus on the carcinogenicity of CBX7 is that CBX7 inhibits the oncosuppressor gene *INK4a/ARF* (Figure 1). For its anti-cancer effect, CBX7 participates in different pathways according to the different tissue environments (Table 1). It mainly interacts with other molecules to epigenetically control the expression of related proteins, thereby regulating some cell signaling pathways to combat cancer manifestations (Such as proliferation, migration, invasion, etc. Figure 2). It needs to be pointed out that different RNA molecules have a complex relationship with CBX7, forming an intricate network between them, making the effect of CBX7 on cancer is not absolute (Figure 3).

CBX7 is involved in the reading recognition of H3K27me3 with its special structure and causes gene silencing, therefore CBX7 may be used as a clinical cancer treatment target. Due to the high degree of homology in the CBX family, antagonists that specifically target the CBX7 reader protein binding to histone methylated lysine have not yet been discovered, but some antagonists have a higher selectivity for CBX7 than CBX2/6/8 (Simhadri et al., 2016). Small molecules such as suramin and Ms37452 can inhibit the binding of lysine methylated polypeptides to the CBX7 chromodomain, which can be used as a feasible cancer suppressor in some cancers with high CBX7 expression, such as prostate cancer (Ren et al., 2015). Furthermore, CBX7 can also increase the sensitivity of cancer cells to chemical drugs (Cacciola et al., 2015; Iqbal et al., 2021). For example, the sensitivity of lung carcinoma cells to irinotecan and the sensitivity of breast cancer cells to FDA-approved drugs. It is worth noting that when defining the function of inhibitory polycomb complexes and evaluating the anti-cancer drugs for CBX7, the overall expression pattern should be given priority, rather than the expression of a single complex member. Of course, the complete mechanism of CBX7 has not been clarified so far. As a subunit of the PcG proteins, CBX7 not only functions independently of PRC1 but also has the function of inhibiting gene expression together with PRC1 as a whole and PCR2 (Scott et al., 2007; Pallante et al., 2015). These phenomena deserve further investigation to elucidate their mechanisms and may provide evidence for the clinical prognostic value and therapeutic targets of cancer.

More importantly, in this review, we briefly illustrated the duality of the interaction between CBX7 and various RNAs in cancers (Figure 3). Similarly, other molecules that use CBX7 as an intermediate for physiological effects can also play a dual role in different cancer progression, such as miR-18a (Wu et al., 2017; Zhao et al., 2020). It is indisputable that the specificity of the environment in which the tissue cells are located affects the duality of CBX7. Additionally, the physical and chemical environment of the tissue may also greatly influence the expression of CBX7 (Chiu et al., 2020). It has been reported that under hypoxic conditions activated hypoxia-inducible factor-1α (HIF-1α) directly binds to the CBX7 gene promoter and activates the expression of CBX7, and hypoxia-induced overexpression of CBX7 stimulates the proliferation of nasopharyngeal carcinoma cells in the ischemic brain (Chiu et al., 2020).

## Conclusion

The most important function of the CBX7 protein is to regulate gene expression by epigenetics. In some cases, it can control cell proliferation, cell apoptosis, and self-renewal. Besides, the cellular effects mediated by CBX7 are diverse in different cancer environments. Therefore, actively guiding the expression of CBX7 *in vivo* according to cancer types may be helpful in guiding the clinical progress of cancer treatment.
